# A pan-cancer analysis of lipid metabolic alterations in primary and metastatic cancers

**DOI:** 10.1038/s41598-023-41107-3

**Published:** 2023-08-23

**Authors:** Guoqing Liu, Yan Yang, Xuejia Kang, Hao Xu, Jing Ai, Min Cao, Guojun Liu

**Affiliations:** 1 School of Life Science and Technology, Inner Mongolia University of Science and Technology, Baotou, China; 2 Inner Mongolia Key Laboratory of Functional Genomics and Bioinformatics, Inner Mongolia University of Science and Technology, Baotou, China

**Keywords:** Cancer, Computational biology and bioinformatics

## Abstract

Metabolic reprogramming is a hallmark of cancers, but pan-cancer level roles of lipid metabolism in cancer development are remains poorly understood. We investigated the possible roles of lipid metabolic genes (LMGs) in 14 cancer types. The results indicate that: (1) there is strong evidence for increased lipid metabolism in THCA and KICH. (2) Although the overall levels of lipid metabolic processes are down-regulated in some cancer types, fatty acid synthase activity and fatty acid elongation are moderately up-regulated in more than half of the cancer types. Cholesterol synthesis is up-regulated in five cancers including KICH, BLCA, COAD, BRCA, UCEC, and THCA. (3) The catabolism of cholesterols, triglycerides and fatty acids is repressed in most cancers, but a specific form of lipid degradation, lipophagy, is activated in THCA and KICH. (4) Lipid storage is enhanced in in kidney cancers and thyroid cancer. (5) Similarly to primary tumors, metastatic tumors tend to up-regulate biosynthetic processes of diverse lipids, but down-regulate lipid catabolic processes, except lipophagy. (6) The frequently mutated lipid metabolic genes are not key LMGs. (7) We established a LMG-based model for predicting cancer prognosis. Our results are helpful in expanding our understanding of the role of lipid metabolism in cancer.

## Introduction

Cancer is the leading cause of human death worldwide. According to the 2020 Global Cancer Statistics, there were approximately 19.3 million new cancer cases and nearly 10 million cancer-related deaths worldwide in 2020^1^. Solid tumor is the product of abnormal proliferation of epithelial cells caused usually by the joint effect of environmental and genetic factors^2^. At the molecular level, cancer was triggered by somatic mutations^3^, the essence of which is the dysregulation of gene expression and relevant oncogenic pathways, thereby resulting in the uncontrolled cell proliferation. In addition to mutations, abnormalities in various gene regulatory factors such as DNA methylation, histone modification, RNA splicing, long-range chromatin interactions, and gene regulatory networks are closely related to cancer formation and development^4,5^.

It has been revealed by increasing evidence that metabolic reprogramming is a crucial driving force of cancer progression^6,7^. Tumor microenvironment varies across individuals and different foci, but tumor cells are able to adapt quickly to the adversity of hypoxia and nutrient deficiency and maintain rapid growth. This adaptation is achieved largely by reprogramming the metabolisms of energy and building blocks of cells^8^. In the past two decades, the roles of numerous lipid metabolic genes as well as cancer signaling genes in lipid metabolic reprogramming were uncovered in various cancers^9–11^. For example, the key enzymes or proteins involved in the lipid metabolic pathways, such as ATP citrate lyase (ACLY), fatty acid synthase (FASN), Acetyl-CoA carboxylase (ACC), Stearoyl-CoA desaturases (SCD), Diacylglycerol acyltransferase (DGAT), fatty acid transporters (CD36), fatty acid-binding protein (FABP), and the sterol regulatory element-binding proteins (SREBPs), were differentially expressed in some cancers^9–11^. The rate-limiting enzyme of gluconeogenesis, phosphoenolpyruvate carboxykinase 1 (PCK1), participated in the activation of SREBP signaling pathway, which was then activate biosynthesis of lipids in human hepatocellular carcinoma (HCC)^12^. In addition, cancer cells preferentially utilize the intermediate of glutamine metabolism, acetyl CoA, for lipid biosynthesis in hypoxia^13^.

Lipids have several functions such as being component of cell membrane, energy source, and signaling molecules. A frequently discussed reprogramming of lipid metabolism in tumor is increased de novo synthesis of lipids^9–11^. Because tumor microenvironment differs greatly between different tumor types, it would inevitably lead to the diversity of tumor lipid metabolism, that is, the inter-tumor heterogeneity of lipid metabolic reprogramming^14^.

Cancer cell metastasis, tumor metabolism, and tumor microenvironment interact with each other. Particular microenvironment enables cancerous cells to alter their metabolic processes to survive and metastasis^15^. Distant metastasis prefers certain organs or tissues such as bone, brain, liver, and lung^16,17^, probably because the microenvironment and metabolic substances exocytosed from stromal cells at metastatic sites favor metastasis. Metabolic reprogramming of primary tumor was also shown to promote cancer cell invasion and metastasis^18^. For example, studies have shown that the up-regulation of cholesterol metabolism in either tumor cells or tumor microenvironment can promote tumor invasion and metastasis. Lipids such as phosphatidic acid and diacylglycerol can act as signaling molecules to promote cancer cell migration^19^. The high expression of a lipase (MAGL) and the fatty acids (FAs) released under its effect of the lipase can promote the migration of cancer cells^20^. The premise of cancer cell metastasis is that cancer cells acquire invasive ability, which is closely related to the process of epithelial-mesenchymal transition (EMT)^21^. At present, little is known about the association between tumor lipid metabolic reprogramming and EMT.

Although a great progress has been made in recent years regarding the role of lipids in cancer development, the inter-tumor heterogeneity of lipid metabolism and the role of lipid metabolic reprogramming in metastasis still remain unclear at pan-cancer level. In this study, we conducted a comprehensive analysis of tumor transcriptome and mutation data, aiming to obtain a general picture of lipid metabolic reprogramming before and after tumor metastasis, and to establish a model for predicting tumor prognosis based on the expression level of lipid metabolic genes.

## Results and discussion

### Abnormal lipid metabolism in primary tumors

**Figure 1:**
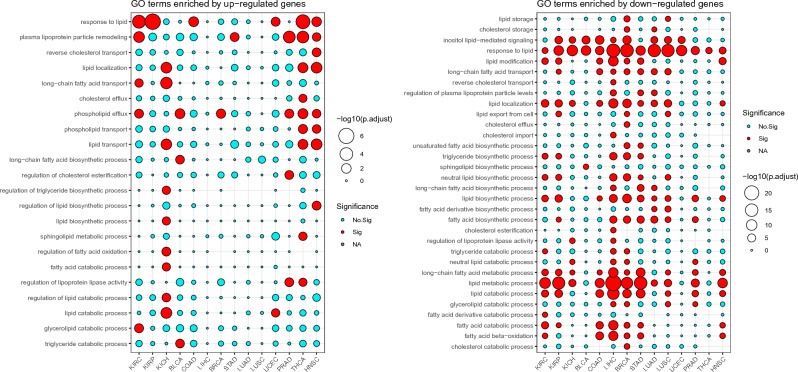
Activities of lipid metabolic processes in primary cancers inferred from GO enrichment analyses over DEGs. Only representative GO terms were manually selected and presented to avoid redundancy in GO terms of similar meaning.

Based on the TCGA-derived gene expression data of 14 primary tumors and normal samples, differential gene expression analyses were conducted. The details of sample information and differentially expressed genes were shown in Supplementary Table S1. What we are interested to see is the enrichment of pathways or GO terms about lipid metabolism. Therefore, we carried out gene set enrichment analyses over the DEGs. We filtered all the lipid metabolism-related enriched GO terms and presented those enriched at least in one cancer type (Fig. 1). The results show that: (1) for most of the cancer types, up-regulated DEGs are rarely enriched with lipid metabolic terms, except for three cancer types including THCA, HNSC and KICH (Fig. 1A). Specifically, both synthetic and catabolic processes are not activated in most of the analyzed tumors. However, it is interesting that both synthetic and catabolic processes are activated in KICH, but not in the other two kidney cancers (KIRC and KIRP). In addition, although THCA and HNSC appear to be dependent on lipid metabolism, it is only limited to elevated lipid transport activity (Fig. 1), suggesting that two cancer types are likely to exchange lipids with their tumor microenvironment. However, we will show evidence supporting the enrichment of lipid biosynthetic process in THCA in GSVA analysis section later. (2) Contrasted with the small number of lipid metabolic GO terms enriched by up-regulated DEGs, there are much abundant lipid metabolic GO terms enriched by down-regulated DEGs (Fig. 1B). A clear trend is that the cancer types with no (or few) enhanced lipid metabolic processes exhibit strongly reduced lipid metabolic processes (enrichment in down-regulated genes, Fig. 1B). For example, lipid biosynthesis, catabolism, and lipid-mediated signaling are reduced in LIHC, BRCA, STAD, LUSC, KIRC and KIRP. The three cancer types (THCA, HNSC, and KICH) do not display remarkable evidence for reduced lipid metabolic processes, and this is inherently consistent with the results presented in Fig. 1A. Note that although DEG-based GO enrichment analysis indicates the down-regulation of the overall level of lipid metabolism including lipid biosynthesis and catabolism, the upcoming analysis of individual lipid metabolic genes suggest an up-regulated biosynthesis of fatty acids, cholesterols, phospholipids, sphingolipids and diacylglycerols in some cancer types (see text results regarding Fig. S1A).

### Gene set variation analysis of primary and metastatic tumors

**Figure 2:**
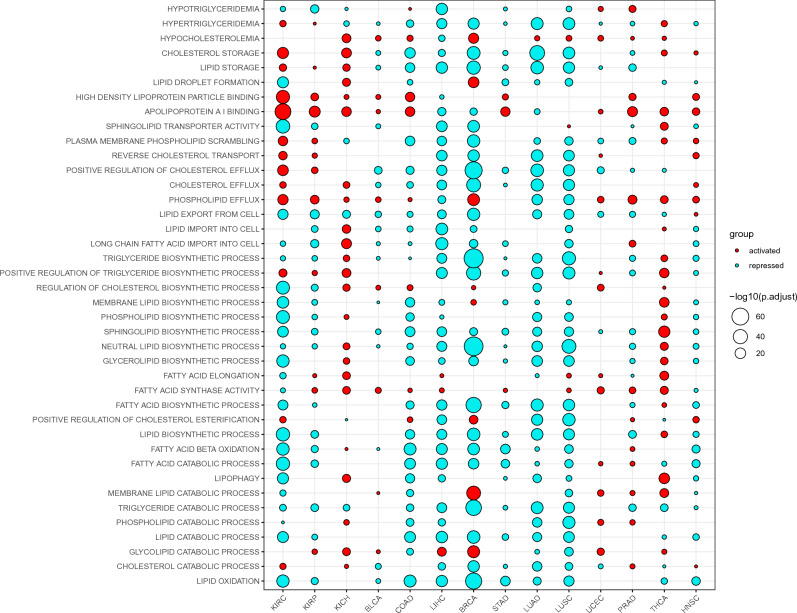
Activities of lipid metabolic processes in primary cancers inferred from GSVA analyses. Only representative GO terms were manually selected and presented.

In order to provide further evidence for the aforementioned results about lipid metabolism in cancer, we also performed gene set variation analysis (GSVA) analysis. We summarized the enrichment of lipid metabolic processes from the following aspects: catabolic process, biosynthetic process, lipid transport, and lipid storage (Fig. 2).

#### Lipid catabolic process

The overall level of lipid catabolism is reduced in almost all cancer types (10 out of 14; Fig. 2). For example, the catabolism of cholesterols, triglycerides and fatty acids is repressed in most cancers. However, the catabolism of glycolipids and membrane lipids is elevated in most of the cancer types, such as BRCA, UCEC, and THCA. The increased catabolism of glycolipids is consistent with the increased requirement of energy source for the fast-proliferating cancer cells. Although catabolism of cholesterols and triglycerides are reduced in most cancer types, a specific form of lipid degradation, lipophagy, is activated in THCA and KICH (Fig. 2). Lipophagy is a process that lipid droplets, where triglycerides and cholesterol esters are stored, are degraded in autophagy vacuole in response to lipid overload or deficit of raw materials required for lipid synthesis^22,23^. Because free fatty acids at high concentration could induce cytotoxicity^24^, excess fatty acids in a cell due to fatty acid uptake or de novo synthesis are stored in the form of cholesterols and triglycerides in lipid droplet^25,26^. Cancer cell can mobilize lipids in droplets in response to starvation to produce fatty acids and then ATP via two pathways: lipolysis and lipophagy. The role of lipophagy in cancer is controversial^27^. For example, autophagy defects in the degradation of lipid droplets promote renal clear cell carcinoma, suggesting the tumor suppressing role of autophagy^28^. On the other hand, lipophagy is able to drive cancer progression by providing cancer cell with fatty acids for ATP production and intermediate substrates for biomolecule synthesis^22,23,27^. Consistent with latter role, our results imply that elevated lipophagy may promote the proliferation and survival of cancer cells of THCA and KICH. Not only lipophagy, but also the biosynthetic processes of lipids are pervasively activated in these two cancer types (THCA and KICH, Fig. 2), suggesting that THCA and KICH are two cancer types that depend strongly on the lipid metabolism.

In addition, β-oxidation of fatty acids is down-regulated across diverse cancer types except for PRAD and KICH, where a slight up-regulation of β-oxidation of fatty acids is observed. The pervasively reduced β-oxidation of fatty acids in cancer implies that many tumors rely preferentially on other routes (e.g. glycolysis), rather than fatty acid β-oxidation, to supply ATP for their growth. It was shown that fatty acid oxidation was used to produce abundant ATP and support triple-negative breast cancer and glioma^29–31^. In addition to these two types of cancer, our results suggest that the ATP demand of PRAD and KICH depends on fatty acid oxidation.

#### Lipid biosynthetic process

Seemingly, fatty acid biosynthetic process is not enriched across diverse cancer types (Fig. 2). However, a closer inspection indicates that fatty acid synthase activity and fatty acid elongation are moderately up-regulated in more than half of the cancer types (Fig. 2). This is consistent with the role of FASN, a fatty acid synthase, in de novo synthesis of fatty acids in human cancers^32^. Synthetic processes of neutral lipids, and complex lipids, such as phospholipids and sphingolipids, are generally down-regulated in cancers (Fig. 2), with two up-regulated exceptions (THCA and KICH). It is biologically inconsistent that fatty acid synthase activity and fatty acid elongation are up-regulated but biosynthesis of fatty acid-based complex lipids is down-regulated. In order to understand more deeply the phenomenon, we manually compiled the crucially important genes involved in lipid metabolism and inspected their expression (Fig. S1). It is possible that the biosynthesis of both phospholipids, sphingolipids and diacylglycerols is selectively up-regulated by specific genes, such as GPAT2 for phospholipid synthesis, SMS for sphingolipid synthesis, and DGAT1/DGAT2 for diacylglycerol synthesis (Fig. S1A). In other words, based on the expression analysis for individual genes, we tend to believe the up-regulation of biosynthesis of both phospholipids, sphingolipids and diacylglycerols in many cancer types, even though it is not supported by GSVA results. In addition, GSVA results show that cholesterol synthesis is up-regulated in five cancers including KICH, BLCA, COAD, BRCA, UCEC, and THCA. Gene expression analysis also suggests the possible genes that are responsible for the increased cholesterol synthesis in KICH, BLCA, BRCA, LUSC and UCEC (Fig. S1). Both GSVA and gene expression analysis consistently support the up-regulation of cholesterol synthesis in KICH, BLCA, BRCA and UCEC.

#### Lipid transport

Lipid uptake is enhanced in THCA and KICH (Fig. 2). Increased lipid uptake, together with the up-regulation of lipid synthesis and lipid droplet degradation in THCA and KICH, suggests again the high lipid demand for these two cancer types. It is unclear why phospholipid efflux is enriched across diverse cancer types (Fig. 2). The gene set enrichment analysis also supports the increased phospholipid efflux in numerous cancers (Fig. 1A). A slight increase in cholesterol efflux is observed in kidney cancers (KIRC, KIRP, and KICH).

#### Lipid storage

Lipid storage is enhanced in in kidney cancers (KIRC, KIRP, and KICH) and thyroid cancer (THCA). Specifically, cholesterol storage is up-regulated in KIRC, KICH, and THCA, and triglycerides storage in KIRC, KIRP, and THCA (Fig. 2). Enrichment of lipid droplet is also detected in BRCA (Fig. 2). Accumulation of LD was reported in kidney cancer^33^, and colorectal cancer^34^, and was shown to relate to cancer aggressiveness^34^ and chemo-resistance^35^. Our results expanded the list of cancers with lipid droplet accumulation.

From the results of gene expression analysis (Fig. S1), we also see that genes involved in lipid degradation (not FA degradation), particularly in the degradation of phospholipids (Fig. S1A: PLA2G7-ALDOC), are up-regulated in numerous cancer types. The degradation of phospholipids would yield some metabolites, such as LPA, that promote cancer cell proliferation in autocrine or paracrine manner through acting as signaling molecules^36^. But the down-regulation of LPA receptor (LPAR1) may render LPA signal ineffective (Fig. S1B). Further study is required to address if and how LPA contribute to cancer development at pan-cancer level. The metabolite of degradation of sphingolipids, S1P, was shown to drive cancer cell growth and survival^37^. Consistent with this, we also observed a universal up-regulation of SPHK1 across diverse cancer types, which catalyzes the S1P production. In a word, the production of sphingolipid metabolites (S1P) plays an important role in cancer development at pan-cancer level.

We also found that the hydrolysis of triglycerides is extremely low in KICH, suggesting the accumulation of TG in KICH. It is worth noting that although GSEA and GSVA analyses based on the overall expression of gene set suggest a reduced catabolic process of triglycerides in almost all cancers analyzed, it is possible that a normal-level or even a slightly increased degradation of triglycerides exists in tumors except KICH, as suggested by the up-regulated expression level of certain TG-associated lipase genes (Fig. S1A). For example, lipases that have triglyceride lipase activity, such as LIPF, LIPG, and so on, are up-regulated in some cancer types. However, one should note that MGLL (Monoglyceride Lipase) which converts monoacylglycerides (MAG) to free fatty acids and glycerol, is down-regulated in diverse cancer types, suggesting that the purpose of TG hydrolysis is to produce DAG or MAG, rather than glycerol and FA. It is understandable that the increase of DAG in cancer cells benefit tumor development by promoting the synthesis of complex lipids such as PE, PC, and PS, because DAG is the prerequisite substance for the biosynthesis of complex lipids.

The gene expression levels of lipid transporters show that: long chain fatty acids are transported into mitochondria primarily using CPT1B and CPT1C, instead of CPT1A; Long chain fatty acids are imported into cancer cells preferentially by FABP5-FABP7. Membrane receptors of LDL and VLDL carrying cholesterols are globally down-regulated in diverse cancers, suggesting that cancer cells do not depend strongly on cholesterol import, and thereby the cholesterol biosynthesis might be the major route to supply cholesterols required for cell membrane building in cancer cells. This inference is also supported by the aforementioned GSVA result (Fig. 2). Two cancer types, LIHC and KICH, apart from biosynthetic process, may also import extracellular cholesterols via up-regulated VLDL for their proliferation (Fig. S1). It was reported that lysophosphatidic acid (LPA) can stimulates cell proliferation, migration and survival by acting on its cognate G-protein-coupled LPA receptors (LPARs)^36,38^. However, receptor of LPA, LPAR1 has reduced expression across all the cancer types, suggesting the cancers analyzed in this study do not subjected to LPA-mediated signaling. CD36 is down-regulated in most cancers, suggestive of reduced lipid uptake and hence increased lipid synthesis for most cancers. The expression levels of genes encoding lipid exporters, such as ABCA1 and ABCG1, are up-regulated in some cancers, which is consistent with aforementioned GSVA results.

We screened out the genes that were consistently up-regulated and down-regulated (|log_2_FC|> 1 and p-adjust < 0.001) in at least 10 of 14 cancer types (Fig. S2). These LMGs may play a potential consistent role in tumor development.

**Figure 3:**
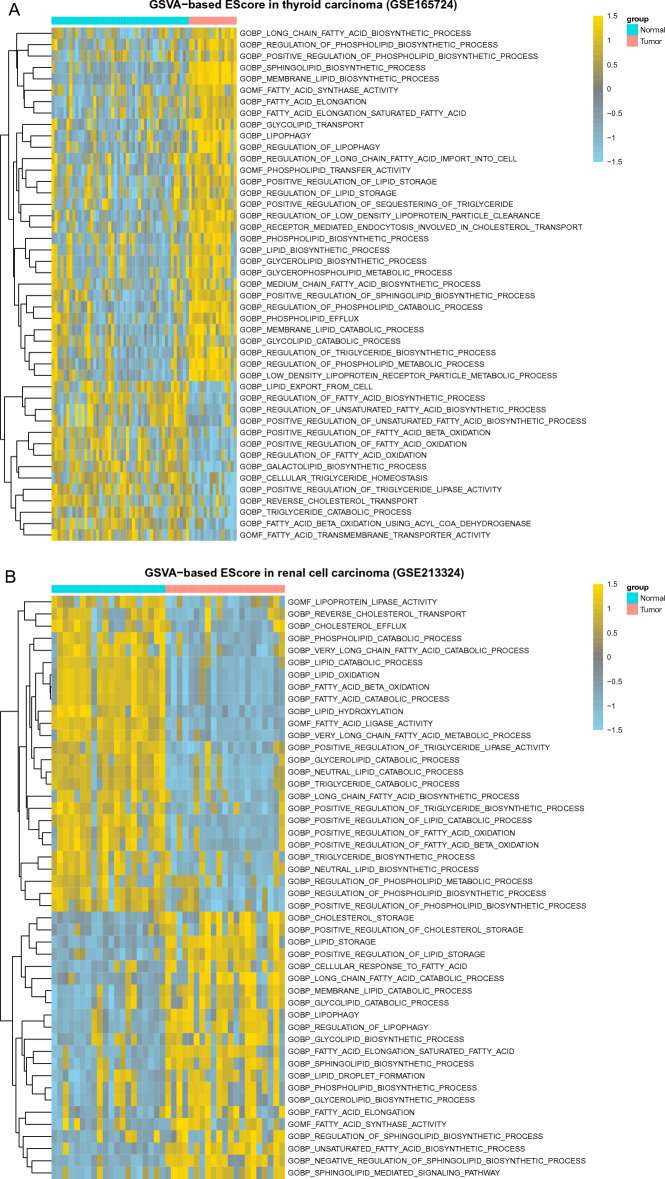
Activities of lipid metabolic processes in two GEO datasets. (**A**) Papillary thyroid carcinoma. (**B**) Renal cell carcinoma. Only representative GO terms were manually selected and presented. Each column represents a sample and each row represents a significant GO term. Heatmap color represents GSVA-based E-score that is used to quantify the activity of biological processes.

Our aforementioned results indicate that lipid metabolism in THCA and KICH are strongly reprogrammed. We further analyzed two GEO datasets (GSE165724 for thyroid cancer, GSE213324 for renal cell cancer) to provide additional supporting evidence for the results. Consistent with our TCGA data-based results, lipid metabolic alterations in thyroid cancer and renal cell cancer were remarkable: (1) in thyroid cancer (Fig. 3A), fatty acid synthase activity, fatty acid elongation, lipophagy, and lipid biosynthetic processes such as sphingolipid biosynthetic process, glycerolipid biosynthetic process, and phospholipid biosynthetic process, are over-activated, while positive regulation of fatty acid beta oxidation, reverse cholesterol transport, and triglyceride catabolic process are down-regulated; (2) In renal cell cancer (Fig. 3B), fatty acid beta oxidation, triglyceride catabolic process, triglyceride biosynthetic process are down-regulated, while fatty acid synthase activity, fatty acid elongation, and diverse lipid biosynthetic processes, lipid droplet formation, cholesterol storage and lipophagy are up-regulated. These results support TCGA data-based conclusion.

**Figure 4:**
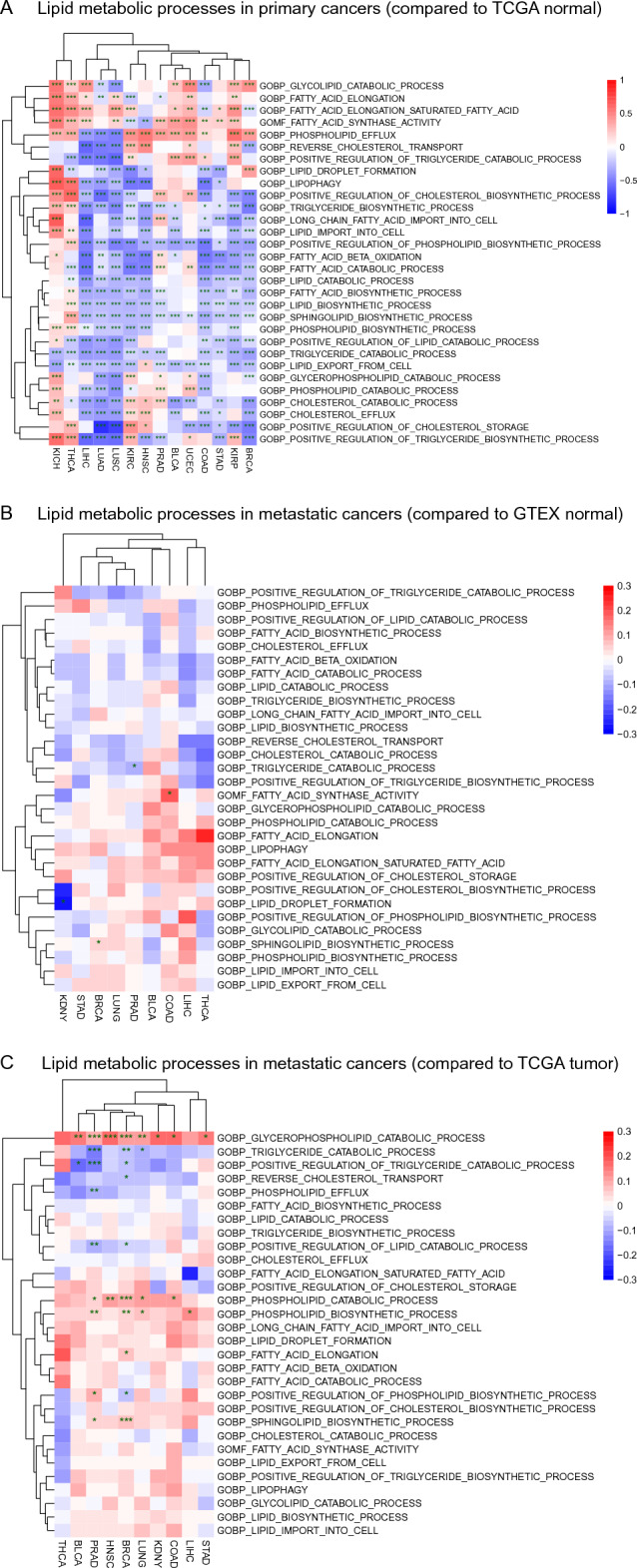
Comparison of lipid metabolic processes between primary and metastatic cancers based on GSVA analyses. Only representative GO terms were manually selected and presented. (**A**) Lipid metabolic processes in primary cancers as compared to normal control. (**B**) Lipid metabolic processes in metastatic cancers as compared to normal control. (**C**) Lipid metabolic processes in metastatic cancers as compared to primary tumor. The heatmap color represents log_2_FC. Significant level of differential expression: ‘*’, ‘**’, and ‘***’ denote p-value < 0.05, < 0.01, and < 0.001, respectively.

To compare lipid metabolic activities between primary and metastatic tumors, we further performed the GSVA on metastatic tumors (MET500 dataset) using GTEX datasets as a normal control. The sample information of the two datasets was shown in Supplementary Table S2. The results show that, in metastatic tumors, there is a global trend of up-regulation in terms of biosynthesis of diverse lipids, while lipid catabolic processes except lipophagy generally tend to be down-regulated (Fig. 4B). Although very similar to those of primary tumors (Fig. 4A), these trends in metastatic tumors are mostly statistically insignificant (Fig. 4), suggesting a greatly alleviated alteration in lipid metabolism in metastatic tumors. Lipid transports between cancer cells and extracellular environment are statistically insignificant as well. The primary tumors harbor abundant differentially expressed LMGs, but metastatic tumors are short of differentially expressed LMGs (Table S2). It is likely that, compared with the primary tumors that adopt various means of lipid metabolic reprograming in severely stressed tumor microenvironment, metastatic tumors analyzed in this study may represent a population whose development depends on lipid metabolism similarly as normal tissues. Similarly, we also evaluated lipid metabolic activities in metastatic tumors directly using primary tumor samples as a control. The results show that phospholipid catabolic process and phospholipid biosynthetic process are upregulated in metastatic tumors, but triglyceride catabolic process is downregulated (Fig. 4C).

### Potential lipid metabolism-related biomarkers of tumor invasion

**Figure 5:**
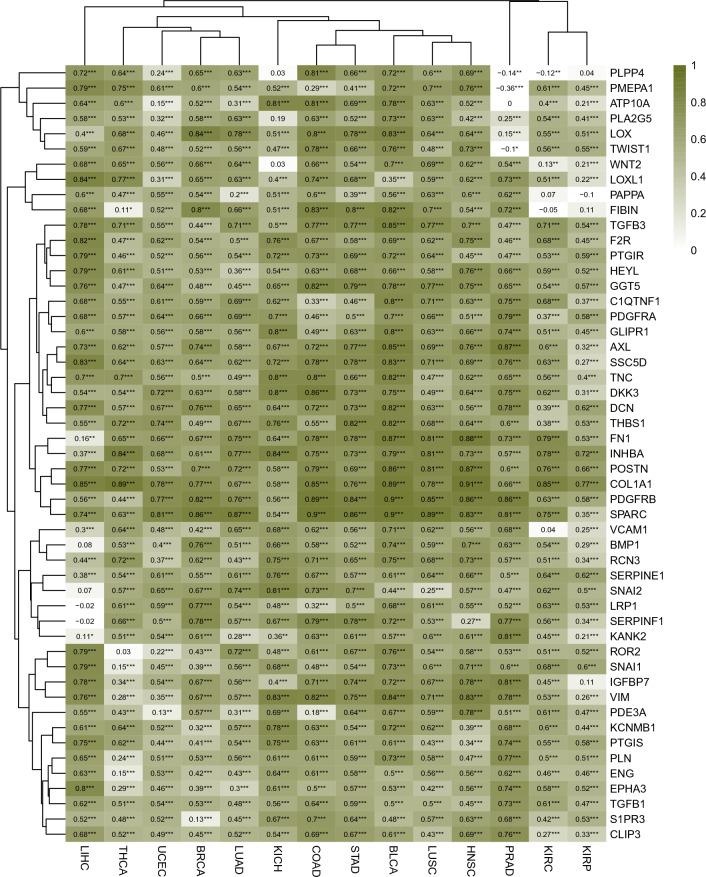
The Spearman correlations between the expression levels of LMGs and GSVA enrichment score of EMT process. Heatmap color represents correlation coefficients. Both correlation coefficients and significant levels (‘*’, ‘**’, and ‘***’ denote adjusted p-value < 0.05, < 0.01, and < 0.001, respectively) are also displayed in the map.

When cancer cells invade and metastasize, the epithelial-derived cancer cells undergo morphological and functional changes and become invasive mesenchymal cells, and this process is called epithelial-mesenchymal transition (EMT)^21^. Assuming that some lipid molecules play an important role in the EMT process of cancer cells, it is highly likely that there is a strong correlation between the expression levels of LMGs and the activity of EMT process. Indeed, some genes involved in lipid metabolism, such as FASN, CAV1, CD36, CPT1C, CYP2E1, and MLXIPL, might be associated with EMT process and cancer cell invasion^39–41^. Furthermore, brain metastasis of breast cancer is assisted by increased de novo synthesis of fatty acids driven by high expression of FASN in the metastasized breast cancer cell^42^. We therefore performed GSVA analyses based on the EMT gene set and gene expression matrix to test the enrichment of EMT process in primary tumors, and then analyzed the correlation (Spearman correlation) between the expression levels of LMGs and the E-Score of the enriched EMT gene sets. From the correlation results, we screened for LMGs strongly correlated with EMT process (correlation coefficient |R|> 0.5, adjusted p value < 0.05) in at least 9 cancer types. As a result, a total of 51 LMGs were identified (Fig. 5). We see that these genes could roughly be classified, according to the biological processes they involved in, into two groups: genes involved in lipid metabolism and genes involved in cellular response to lipids. Among them, some genes are well-known EMT markers. For example, COL1A1 promotes EMT process by expressing and secreting type I collagen that was known to constitute ECM. In fact, what we really interested in is LMGs that could directly promote the EMT process or indirectly stimulate the ECM-related genes like COL1A1. Hence, further study is required to test which of the 51 LMGs are really the lipid-related drivers of EMT process. In addition, based on GSVA-based EMT scores, we compared the EMT activity between metastatic and primary tumors by using limma. No significant difference was detected (Table S3).

### Analysis of mutation spectrum of primary cancer

**Figure 6:**
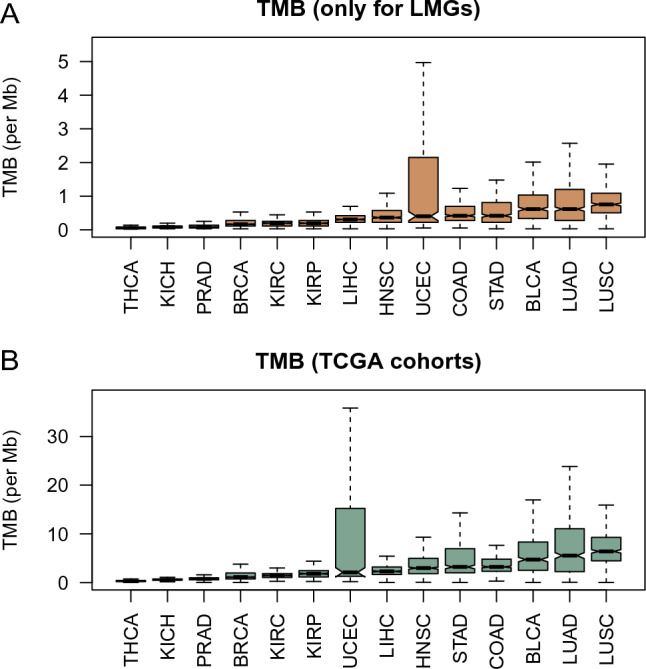
Tumor mutation burden (TMB) across diverse cancer types. (**A**) TMB is calculated by solely using LMG mutations. (**B**) TMB is derived from TCGA cohorts.

We performed mutational profiling analysis on 14 cancers and obtained the TMB of each cancer. TMB reflects the degree of mutation in the tumor cell genome. Tumor patients with high TMB have the potential to acquire more neoantigens and are associated with intratumor heterogeneity. Studies have also shown that patients with high TMB is associated with high immunotherapy benefit. Our first focus regarding mutation is: whether the TMBs derived solely for LMGs is consistent with overall TMB of TCGA cohorts. Our results show that, for both LMG-derived TMB and overall TMB of TCGA cohorts, lung squamous cell carcinoma (LUSC) has the highest TMB, while thyroid carcinoma has the smallest TMB (Fig. 6). Furthermore, despite the slight difference in the rank of TMB of UCEC between Fig. 6A,B, the ranking of LMG-derived TMBs across the different cancer types is largely consistent with that of TCGA cohorts, suggesting that the factors affecting mutation numbers of LMGs across diverse cancer types act similarly to that of genome-level mutation numbers.

**Figure 7:**
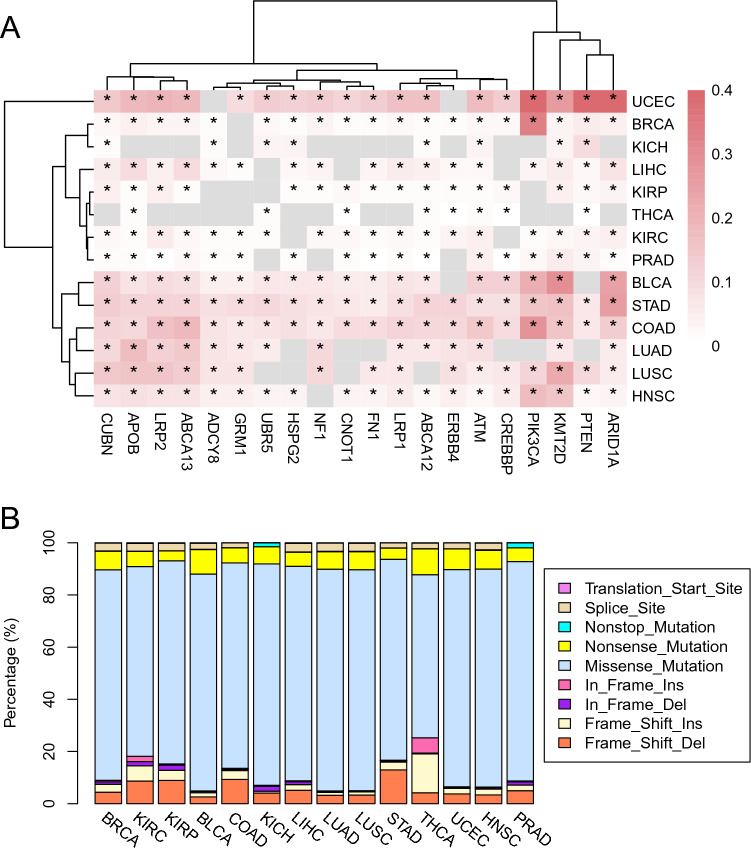
Comparison of mutations in LMGs across diverse cancer types. (**A**) Twenty LMGs with high mutation frequency and are mutated in at least 10 of the 14 tumor types. Heatmap color represents the percentage of mutated samples. The star symbol represents mutation of the gene in the corresponding cancer, while the gray without star symbol means the gene is not in the top 80 mutated genes. (**B**) Percentages of diverse mutation types in cancer.

To explore the potential relationship between mutations and the lipid metabolic reprogramming, we screened for the top 80 LMGs with the highest mutation frequency (percentage of mutated samples) in each cancer, and then selected from them the LMGs that were mutated in at least 10 of the 14 tumor types, resulting in a total of 20 LMGs (Fig. 7A). A simple hypothesis is that the lipid metabolic genes regulate lipid metabolism in tumors via utilizing frequent mutations or avoiding mutations, which in turn affects the occurrence and development of cancer. The results show that the frequently mutated lipid metabolism-related genes are not key LMGs that play direct and critical roles in lipid metabolism, such as FASN and ACSL, but tend to be those involved in cancer-promoting signaling pathways (Fig. 7A). We also observed that the two cancer types, KICH and THCA, which were shown to depend strongly on lipid metabolism, had fewer mutations in their LMGs (Fig. 7A).

Among the top 80 lipid metabolic genes with the highest mutation frequency, a gene, ATM, was found to be mutated across all tumors considered in this study. Through the GeneCards (https://www.genecards.org) database query, it was found that ATM is related to the regulation of cell cycle checkpoint and plays a central role in DNA damage repair. In addition, we observed that among the mutations of LMGs, the missense mutation was the most abundant, followed by the nonsense mutation and frame shift deletion (Fig. 7B). In particular, apart from the missense mutation, the insertional mutations, including the frame shift insertion and in-frame insertion, are relatively high in thyroid cancer (Fig. 7B, THCA).

### Lipid metabolic genes are predictive of cancer survival rate

We explored the relationship between expression levels of differentially expressed LMGs and the survival rate (see “Materials and methods” section for details). A risk score was calculated for each cancer sample based on the expression levels of differentially expressed LMGs, and samples were divided into two groups according to the risk score. Kaplan–Meier survival analysis was then performed to evaluate the overall effect of multi-LMGs on survival.

**Figure 8:**
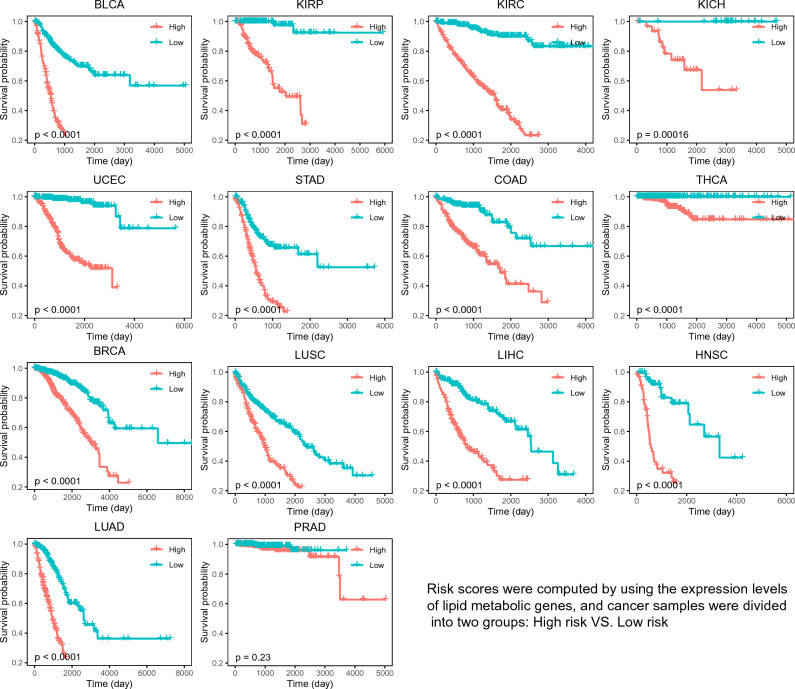
Kaplan–Meier survival rate comparison between two groups of risk scores computed by integrating expression values of multi-LMGs in multivariate Cox survival analysis.

The results show that the survival rates for most cancer types are strongly dependent on the expression level of LMGs (Fig. 8), suggesting again that altered lipid metabolism in cancer affect cancer development and prognosis. The survival rates for kidney cancer (KIRC, KIRP, KICH), bladder cancer (BLCA) and Uterine Corpus Endometrioid Carcinoma (UCEC) are highly associated with the risk score calculated on the LMG expression. It is worth mentioning that, despite some LMGs affect survival for prostate cancer (PRAD), the overall effect of lipid metabolic genes on prostate cancer survival is the slightest (Fig. 8). The list of LMGs used to establish the risk score model (see method section) and regression coefficients (${\beta }_{i}$) of the LMGs were provided at https://github.com/gqliu1010/PanCancer_lipid. In addition, we developed a user-friendly webserver using the R Shiny package for assessing the relationship between the expression levels of LMGs and cancer survival, which was freely accessible at https://whaoe.shinyapps.io/KMSurvival_shiny/. Kaplan–Meier survival analyses based on single LMG expression level or risk score calculated by integrating multi-LMGs can be done on the webserver. In order to provide a concise and crucial survival-related LMG list, we also filtered out the LMGs that were significantly associated with survival (Kaplan–Meier or univariate Cox survival analysis) at least in 5 cancer types, and the top three survival-related LMGs (Kaplan–Meier survival analysis) for each cancer type (Fig. S3).

### Concluding remarks

The results presented above are all based on RNA-seq data. In order to provide protein-level supports for our RNA-level results, we selected some lipid metabolic genes which are on our candidate list (Figs. S1, S2, S3) that may affect cancer development, and defined a weighted index (see “Materials and methods” section) to give approximate estimates of corresponding protein expression levels based on the immunohistochemistry results stored in HPA databank (https://www.proteinatlas.org/). The results indicate that the most of the analyzed genes, such as FASN, SPHK1, CERS1, E2F1, CA9 and SPP1, exhibited a largely consistent protein-level expression with the RNA-level expression (Fig. S4).

In this study, we focused on the behaviors of lipid metabolism at Pan-cancer level, aiming to give a global picture of lipid metabolism in cancers and provide a list of lipid metabolic genes that may play roles in cancer development and survival. Our next goal is to uncover the more specific mechanisms and roles of the altered lipid metabolism in cancer development by focusing on a particular cancer type, such as thyroid cancer or kidney cancer. An important question in this regard is the interaction between lipid metabolism of cancer cells and tumor microenvironment. Single cell sequencing data is required to confirm the relationship between tumor lipid metabolism and microenvironment, and we hope put our much effort into this in the future.

Transcriptomic and genomic data of the 14 cancer types in TCGA were analyzed in this study. Our results indicate that THCA and KICH are characterized by increased lipid biosynthesis, degradation, and import of extracellular lipids, suggesting that the development of the two cancer types depends strongly on the lipid metabolism. Although the overall level of lipid metabolic activity were shown to be reduced in many cancer types including LIHC, BRCA, STAD, LUSC, KIRC and KIRP, we also observed some evidence for moderate up-regulation of the biosynthesis of fatty acids, cholesterols, phospholipids, sphingolipids and diacylglycerols in some cancer types. Lipid storage is enhanced in in kidney cancers (KIRC, KIRP, and KICH) and thyroid cancer (THCA). Metastatic cancers behave similarly to corresponding primary cancers in terms of lipid metabolism as aforementioned. The frequently mutated lipid metabolic genes are not key lipid metabolic genes but tend to be genes that indirectly regulate lipid metabolism or cancer-promoting signaling pathways. Two cancer types, KICH and THCA, which were shown to depend strongly on lipid metabolism, had fewer mutations in their LMGs. These mutational signatures further support the importance of lipid metabolism in the cancers. We also identified some lipid metabolism-associated biomarkers of cancer cell invasion, and established prognostic model based on the expression level of lipid metabolic genes.

In a word, we revealed comprehensive details of lipid metabolic signatures in 14 cancer types in terms of lipid catabolism, lipid biosynthesis, lipid storage, and lipid transport. Although some lipid metabolic alterations are shared across diverse types of cancer, our results highlighted the high inter-tumor heterogeneity and flexibility of lipid metabolism. It is expected that the results presented in this study would provide a deeper insight into lipid metabolic reprogramming of cancer and a candidate list of LMG biomarkers for cancer diagnosis and therapy.

## Materials and methods

### Data acquisition

Transcriptome (level-3 HT-count data), genomic mutation (mutect pipeline), and clinical data of primary tumors were obtained from the TCGA database (https://portal.gdc.cancer.gov/). 14 cancer types with tumor samples and normal samples larger than 19 were investigated in this study (Table S1). Transcriptome data (FPKM format) for metastatic tumors (referred to as MET500) was taken from the study of Robinson et al.^43^, which was available in the UCSC Xena database (https://ucscpublic.xenahubs.net). MET500 consists of whole exome and transcriptome sequencing data of metastasis foci of 22 tissues (or organs) in 500 tumor patients. From MET500, we selected metastatic tumors with the same type of primary tumor origin as primary cancer types obtained from the TCGA database. The gene expression data (FPKM format) of normal tissues obtained from the GTEx database was used as a control (www.gtexportal.org) in differential gene expression analysis for metastatic tumors. Two additional GEO datasets (GSE165724 for thyroid cancer, GSE213324 for renal cell cancer) were also analyzed in this study. From GSE165724, we used gene expression data of normal appearing thyroid tissue adjacent to the tumor samples (n = 46) and paired papillary thyroid carcinoma samples (n = 16). GSE213324 includes 21 renal cell carcinoma samples and 20 normal controls.

Lipid metabolism-related gene sets were obtained from the C5 Ontology Gene Sets obtained from the MSigDB database (http://www.gsea-msigdb.org/gsea/msigdb). To be specific, we chose and integrated all gene sets containing the keywords "lipid", "fatty acid", "lipoprotein", "cholesterol", "triacyglycerol", and "triglyceride". There are 2610 unique genes in the list. The list of key lipid metabolic genes was curated manually based on our literature review and can be partially found in the supplementary file of our previous study^44^. The complete list of lipid metabolism-related genes used in this study can be accessed at https://github.com/gqliu1010/PanCancer_lipid.

### Differential expression analysis

For TCGA-derived data of primary tumors, we performed differential gene expression analysis by using DEseq2^45^. Differentially expressed genes (DEG) were defined as those meeting two criteria: |log_2_FC|> 1 and p-adjust < 0.05, where FC denotes fold change. For metastatic tumors (MET500), gene expression data of normal tissues derived from GTEx database was used as normal control. ComBat^46^ was used to remove the possible batch effect between MET500 and GTEx data, and ballgown package was used for differential expression analysis.

### Gene set enrichment analysis

GO enrichment analysis was performed by using clusterProfiler^47^ separately for up-regulated DEGs and down-regulated DEGs. In addition, gene set variation analysis (GSVA) was performed by using GSVA (R package)^48^. GSVA is a gene set-based differential expression analysis and can generate the enrichment score (EScore) of any gene set of interest. The significance of gene set enrichment (tumor vs. normal) in the GSVA analysis was evaluated by using limma. In the GSVA analysis of TCGA-based primary cancers, DESeq2-based normalized gene expression data were used, and in the GSVA analysis of metastatic cancers, ComBat-based normalized gene expression data were used. For two additional GEO datasets (GSE165724 for thyroid cancer, GSE213324 for renal cell cancer), we carried out GSVA analyses. For GSE165724, raw count data was obtained from GEO, and DESeq2-based normalized gene expression data was used in GSVA analysis. For GSE213324, the already normalized gene expression data available at GEO was used in GSVA analysis.

### Mutational analysis

Based on the TCGA genomic mutation data, the maftools (R package) was used to analyze the mutations of LMGs. In order to compare the mutation abundance for diverse cancer types between LMGs and all genes, LMG-based tumor mutational burden (TMB) was calculated by solely using mutations occurred in LMGs. Pre-computed TMB values for TCGA cohorts were obtained by using maftools.

### Lipid metabolism-based cancer risk model

For primary tumors, lipid metabolism-based survival analysis was conducted by combining Kaplan–Meier survival analysis^49^ and Cox proportional hazards regression model^50,51^. Firstly, differentially expressed lipid metabolic genes (LMGs) were selected. In order to focus on more important LMGs, we used more stringent criteria here for differential expression (|log_2_FC|> 2 and p-adjust < 0.001). Secondly, differentially expressed LMGs were subjected to univariate Cox regression analysis. Thirdly, using multivariate Cox regression^50,51^, a cancer risk score model was established for each cancer type based on the LMGs significantly associated with cancer survival in the second step (p-value < 0.05). The risk score model was formulated as $RiskScore={\sum }_{i=1}^{k}{\beta }_{i}{X}_{i}$, where the ${X}_{i}$ was the expression level of *i*th LMG in the Cox hazards model, and ${\beta }_{i}$ was corresponding regression coefficient. The larger the risk score value, the greater the survival risk of the patient (lower survival rate and worse prognosis). Finally, cancer samples for each cancer type were divided into two groups (high risk and low risk) according to risk scores with a threshold of median risk score, and the survival difference between the two groups was analyzed by using Kaplan–Meier survival analysis^49^.

### Protein expression levels of some lipid metabolic genes

In order to evaluate the protein-level expression of some selected lipid metabolic genes which may affect cancer development, we defined a weighted index based on the immunohistochemistry staining results stored in HPA databank (https://www.proteinatlas.org/). Each protein has multiple staining images and some proteins were stained with more than one antibody. In case of more than one antibody used for a protein, we selected staining results of a particular antibody possessing the largest number of staining samples. The expression level of a protein was denoted as “not detected”, “low”, “medium”, or “high” in the HPA. In order to give an approximate estimate of average expression level of a protein, we assigned scores of 0, 0.33, 0.66 and 1 to “not detected”, “low”, “medium”, and “high” staining respectively, and then defined a weighted expression level index as: $Elevel=\frac{{\sum }_{i=1}^{4}{n}_{i}{Score}_{i}}{{\sum }_{i=1}^{4}{n}_{i}}$, where ${Score}_{i}$ represents the score of immunohistochemistry staining level (e.g. 0, 0.33, 0.66 and 1), and ${n}_{i}$ represents the number of staining images with the corresponding staining level.

### Ethics statement

Ethical review and approval was not required for the study on human participants in accordance with the local legislation and institutional requirements. Written informed consent for participation was not required for this study in accordance with the national legislation and the institutional requirements.

### Supplementary Information

Supplementary Information.

## Data Availability

Publicly available cancer datasets were analyzed in this study, which can be freely downloaded from the TCGA data portal (https://portal.gdc.cancer.gov/). Processed data regarding LMG list, gene differential expression, GSVA analysis, and survival analysis were provided at github (https://github.com/gqliu1010/PanCancer_lipid/branches).

## Abbreviations

DEG: Differentially expressed gene
LMG: Lipid metabolic gene
FA: Fatty acid
FAs: Fatty acids
LDs: Lipid droplets
EMT: Epithelial mesenchymal transition
BLCA: Bladder urothelial carcinoma
BRCA: Breast invasive carcinoma
COAD: Colon adenocarcinoma
HNSC: Head and neck squamous cell carcinoma
KICH: Kidney chromophobe
KIRC: Kidney renal clear cell carcinoma
KIRP: Kidney renal papillary cell carcinoma
LIHC: Liver hepatocellular carcinoma
LUAD: Lung adenocarcinoma
LUSC: Lung squamous cell carcinoma
PRAD: Prostate adenocarcinoma
STAD: Stomach adenocarcinoma
THCA: Thyroid carcinoma
UCEC: Uterine corpus endometrial carcinoma
