# Supplementary material for: A pan-cancer analysis of lipid metabolic alterations in primary and metastatic cancers
Source: Sci Rep. 2023 Aug 23;13:13810. doi: 10.1038/s41598-023-41107-3 (PMC10447541; doi:10.1038/s41598-023-41107-3)
Supplement: Supplementary file 1 — Supplementary Information. [file 41598_2023_41107_MOESM1_ESM.docx]

Supplementary information

A pan-cancer analysis of lipid metabolic alterations in primary and metastatic cancers

Guoqing Liu^*^, Yan Yang, Xuejia Kang, Hao Xu, Jing Ai, Min Cao, Guojun Liu^*^

*** Correspondence:** Guoqing Liu, [gqliu1010@163.com](mailto:gqliu1010@163.com); Guojun Liu, gjliu0325@gmail.com

## Captions for supplementary figure and tables:

**Figure S1.** Expression levels of representative key genes involved in lipid metabolism in primary cancers. (A), Genes involved in lipid metabolic processes. (B), Genes encoding lipid transporters and some other genes of interest. The heatmap color represents log_2_FC, whose values are also displayed in the map. Significant level of differential expression: ‘*’, ‘**’, and ‘***’ denote adjusted p.value<0.05, <0.01, and <0.001, respectively.

**Figure S2.** Consistently up or down-regulated LMGs in primary cancers. The heatmap color represents log_2_FC.

**Figure S3.** Representative survival-related LMGs. (A), Genes are significantly associated with survival (Kaplan-Meier or univariate Cox survival analysis) at least in 5 cancer types. (B), Top three survival –related LMGs with the smallest p-values. The heatmap color represents p-values of survival analyses. The cells in gray denote non-significant. The star ‘*’ in the figure A represent significantly related genes, and star ‘*’ in the figure B represent top 3 genes for each cancer type.

**Figure S4.** Protein expression levels of some LMGs evaluated according to the immunohistochemistry results stored in HPA databank (<https://www.proteinatlas.org/>). See methods section in the main text for expression level estimates.

**Table S1.** Statistical summary of TCGA samples used in this study and differentially expressed genes in tumor

**Table S2.** Statistical summary of metastatic samples used in this study and differentially expressed genes in tumor

**Table S3.** The results of limma-based test of the difference in EMT activity between metastatic tumors and primary tumors

A





B





**Figure S1.** Expression levels of representative key genes involved in lipid metabolism in primary cancers. (A), Genes involved in lipid metabolic processes. (B), Genes encoding lipid transporters and some other genes of interest. The heatmap color represents log_2_FC, whose values are also displayed in the map. Significant level of differential expression: ‘*’, ‘**’, and ‘***’ denote adjusted p.value<0.05, <0.01, and <0.001, respectively.


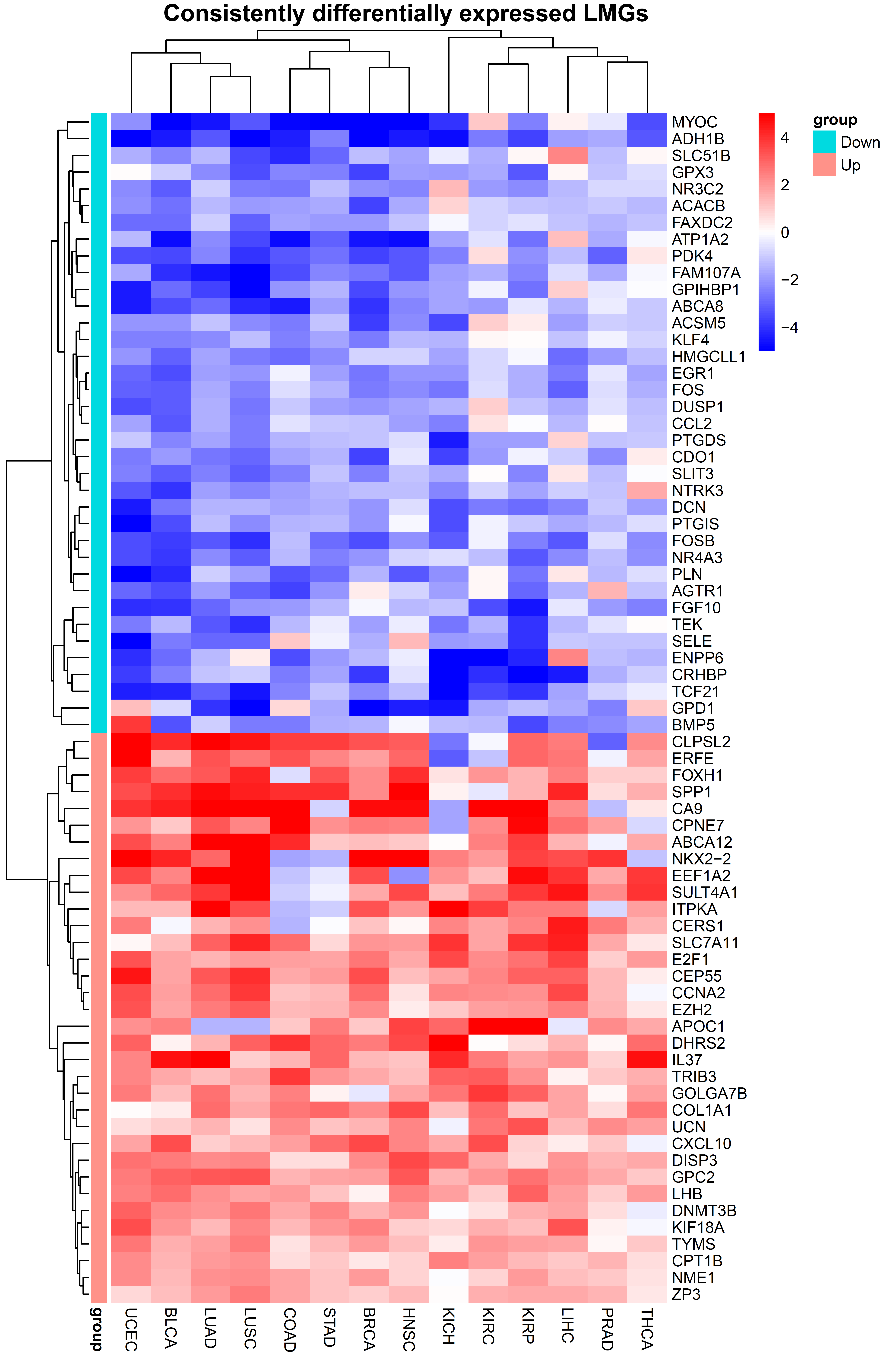


**Figure S2.** Consistently up or down-regulated LMGs in primary cancers. The heatmap color represents log_2_FC.


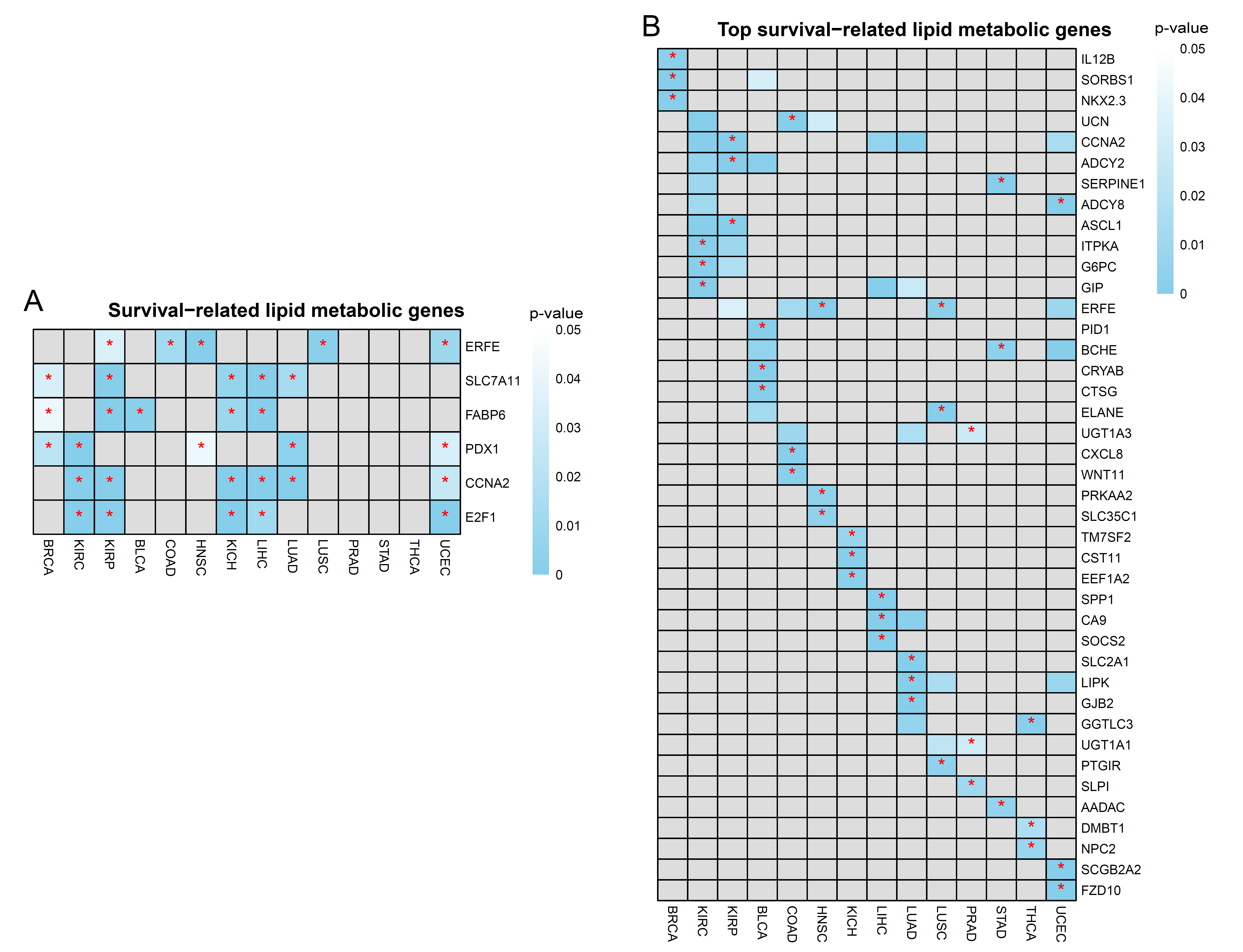


**Figure S3.** Representative survival-related LMGs. (A), Genes are significantly associated with survival (Kaplan-Meier or univariate Cox survival analysis) at least in 5 cancer types. (B), Top three survival –related LMGs with the smallest p-values. The heatmap color represents p-values of survival analyses. The cells in gray denote non-significant. The star ‘*’ in the figure A represent significantly related genes, and star ‘*’ in the figure B represent top 3 genes for each cancer type.


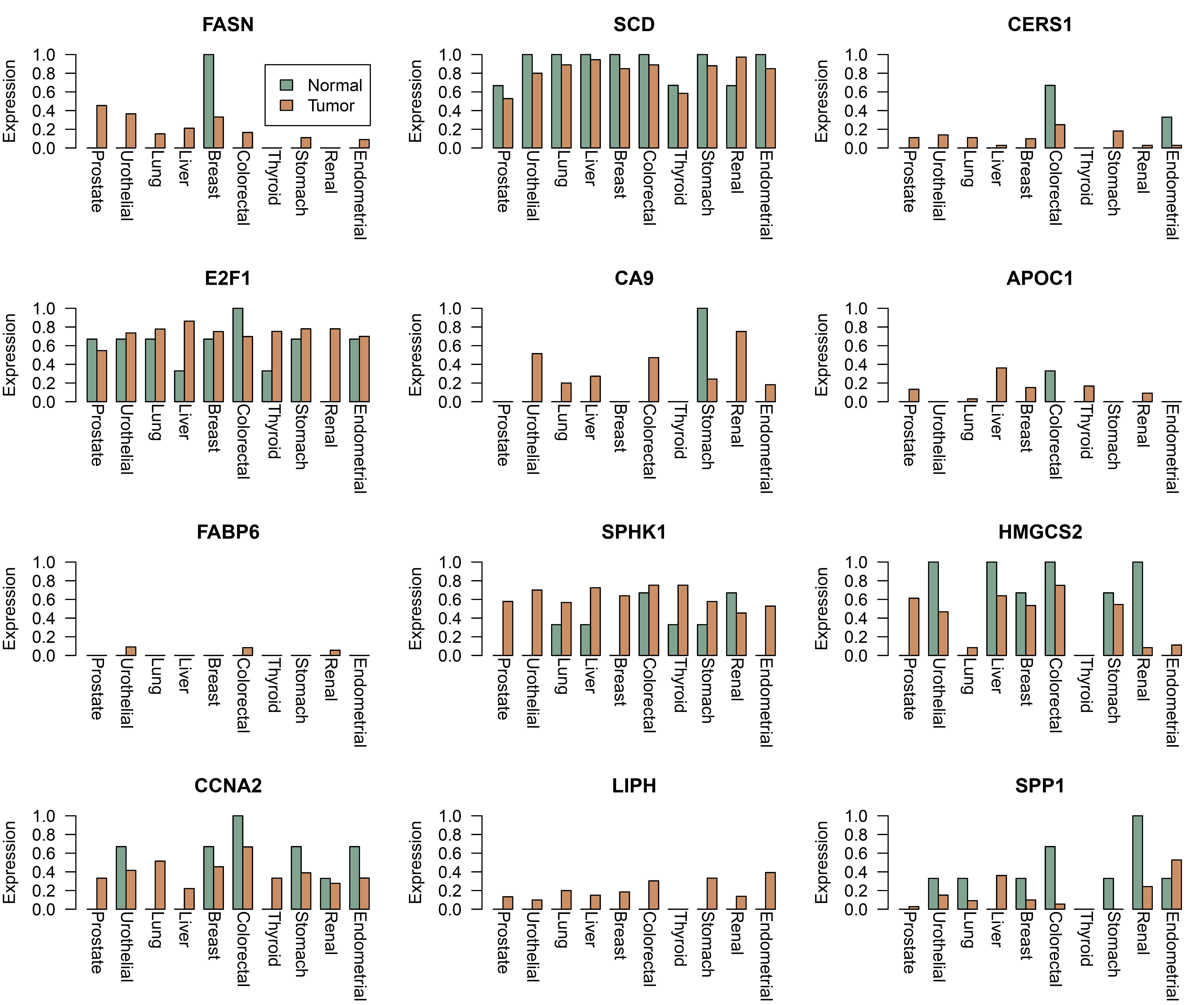


**Figure S4.** Protein expression levels of some LMGs evaluated according to the immunohistochemistry results stored in HPA databank (<https://www.proteinatlas.org/>). See methods section in the main text for expression level estimates.

**Table S1.** Statistical summary of TCGA samples used in this study and differentially expressed genes in tumor

| Cancer type | #NO. tumor samples | #NO. normal samples | #NO. up-regulated genes | #NO. down-regulated genes |
| --- | --- | --- | --- | --- |
| PRAD | 499 | 52 | 1228 | 1716 |
| UCEC | 552 | 23 | 3506 | 2436 |
| BLCA | 414 | 19 | 2245 | 1865 |
| HNSC | 111 | 12 | 2470 | 1448 |
| LUAD | 535 | 59 | 3377 | 1946 |
| LUSC | 502 | 49 | 4100 | 2994 |
| LIHC | 374 | 50 | 3151 | 1202 |
| KIRC | 539 | 72 | 3846 | 2023 |
| KIRP | 288 | 32 | 2658 | 2185 |
| KICH | 65 | 24 | 2463 | 3351 |
| BRCA | 1109 | 113 | 3006 | 2008 |
| STAD | 375 | 32 | 1915 | 2268 |
| THCA | 510 | 58 | 1856 | 1274 |
| COAD | 480 | 41 | 2759 | 2525 |

Note: differentially expressed genes were identified using DESeq2 with criteria |log2FC|>1 and p.adj<0.05

**Table S2.** Statistical summary of metastatic samples used in this study and differentially expressed genes in tumor

| Cancer type | #NO. metastatic samples (MET500) | #NO. normal samples (GTEx) | #NO. up-regulated genes | #NO. down-regulated genes |
| --- | --- | --- | --- | --- |
| PRAD | 155 | 100 | 279 | 232 |
| BLCA | 30 | 9 | 80 | 69 |
| HNSC | 45 | - | - | - |
| LUNG | 42 | 281 | 148 | 644 |
| LIHC | 10 | 110 | 81 | 114 |
| KDNY | 12 | 28 | 197 | 1195 |
| BRCA | 159 | 179 | 376 | 251 |
| STAD | 15 | 174 | 99 | 1915 |
| THCA | 5 | 279 | 247 | 338 |
| COLO | 20 | 308 | 367 | 2495 |

Note: differentially expressed genes were identified using ballgown with criteria p.adj<0.05

**Table S3.** The results of limma-based test of the difference in EMT activity between metastatic tumors and primary tumors

|  | logFC | AveExpr | t | P.Value |
| --- | --- | --- | --- | --- |
| BLCA | 0.023263 | -0.00226 | 0.49411 | 0.621459 |
| BRCA | 0.016929 | 0.015515 | 0.692625 | 0.488669 |
| COLON | 0.056842 | -0.00378 | 0.827983 | 0.408042 |
| HNSC | 0.018034 | -0.00527 | 0.450799 | 0.652294 |
| KDNY | -0.02493 | 0.009498 | -0.26391 | 0.791903 |
| LIHC | 0.056569 | -0.00687 | 0.709489 | 0.478416 |
| LUNG | 0.028107 | -0.00941 | 0.742682 | 0.457831 |
| PRAD | 0.005334 | -0.0016 | 0.232375 | 0.816316 |
| STAD | 0.010143 | 0.005567 | 0.124726 | 0.900792 |
| THCA | 0.010806 | 0.000737 | 0.078426 | 0.937517 |
